# Effects of the Extremely Low Frequency Electromagnetic Fields on NMDA-Receptor Gene Expression and Visual Working Memory in Male Rhesus Macaques

**DOI:** 10.29252/NIRP.BCN.9.3.167

**Published:** 2018

**Authors:** Masoomeh Kazemi, Hedayat Sahraei, Hamed Aliyari, Elaheh Tekieh, Mehdi Saberi, Hassan Tavacoli, Gholam Hossein Meftahi, Hossein Ghanaati, Maryam Salehi, Mostafa Hajnasrollah

**Affiliations:** 1. Neuroscience Research Center, Baqiyatallah University of Medical Sciences, Tehran, Iran.; 2. Faculty of Electrical, Biomedical and Mechatronics Engineering, Qazvin Branch, Islamic Azad University, Qazvin, Iran.; 3. Department of Pharmacology, School of Medicine, Baqiyatallah University of Medical Sciences, Tehran, Iran.; 4. Medical Imaging Centre, Imam Khomeini University Hospital, Tehran, Iran.; 5. Reproductive Biomedicine Research Center, Royan Institute for Biotechnology, Academic Center for Education, Culture and Research (ACECR), Tehran, Iran.

**Keywords:** ELF, Hippocampus, Melatonin, MRI, NMDA receptors, Visual working memory, Rhesus monkey

## Abstract

**Introduction:**

The present research aimed to examine Visual Working Memory (VWM) test scores, as well as hormonal, genomic, and brain anatomic changes in the male rhesus macaques exposed to Extremely Low Frequency Magnetic Field (ELF-MF).

**Methods:**

Four monkeys were exposed to two different ELF-MF frequencies: 1 Hz (control) and 12 Hz (experiment) with 0.7 μT (magnitude) 4 h/d for 30 consecutive days. Before and after the exposure, VWM test was conducted using a coated devise on a movable stand. About 10 mL of the animals’ blood was obtained from their femoral vain and used to evaluate their melatonin concentration. Blood lymphocytes were used for assaying the expressions of N-Methyl-D-aspartate NMDA-receptor genes expression before and after ELF exposure. Anatomical changes of hippocampus size were also assessed using MRI images.

**Results:**

Results indicated that VWM scores in primates exposed to 12 Hz frequency ELF increased significantly. Plasma melatonin level was also increased in these animals. However, these variables did not change in the animals exposed to 1 Hz ELF. At last, expression of the NMDA receptors increased at exposure to 12 Hz frequency. However, hippocampal volume did not increase significantly in the animals exposed to both frequencies.

**Conclusion:**

In short, these results indicate that ELF (12 Hz) may have a beneficial value for memory enhancement (indicated by the increase in VWM scores). This may be due to an increase in plasma melatonin and or expression of NMDA glutamate receptors. However, direct involvement of the hippocampus in this process needs more research.

## Highlights

- 12-Hz ELF exposure enhances the brain cognitive function.
- Brain cognitive function enhancement may be due to increase in plasma melatonin hormone and or increase in NMDA glutamate receptor gene expression.

## Plain Language Summary

Extremely Low-Frequency Electromagnetic field is one of the most important factors affecting the environment. This study aimed to determine the effects of 12-Hz frequency field on the visual changes of male monkeys. The research findings, because of the cognitive similarity of the monkeys to humans, can be generalized to humans. The results showed that the ELF/EMF increased the expression of the NMDA -R genes and melatonin hormone. They have two important roles in improving visual working memory. This study demonstrates the effect of the 12-Hz frequency on the monkey’s visual memory. Researchers can use 12- Hz frequency on other cognitive indices.The test frequency may increase alpha brain waves (12 Hz), however, its confirmation requires more research.

## 1. Introduction

Numerous studies have been carried out to reveal the biological, physiological, and behavioral effects of electromagnetic fields on humans and animal models. Given the biological similarities between monkeys and humans, more explicit and comprehensive studies have been carried out on the monkeys, especially Macaca mulatta species known as the rhesus macaque (De Lorge, & Grissett, 1977; Fabbri-Destro & Rizzolatti, 2008; Mitchell & Leopold, 2015) which their genes have 98% similarity with humans (Baharara & Zahedifar, 2012; Fang et al., 2011; Kanthaswamy et al., 2013). Extremely Low Frequency (ELF) electromagnetic fields ranging from 1 to 300 Hz induce different effects on living organisms (Tekieh et al., 2017; Zhu et al., 2016). In this regard, researchers have examined the effect of electromagnetic fields on important biological processes such as cell proliferation, ion exchanges across the biological membranes, bone repair, nerve repair, production of free radicals, hormonal changes, enzyme activity modulation, and changes of membrane and intracellular proteins (Al-Akhras, Darmani, & Elbetieha, 2006; Kula, Sobczak, & Kuska, 2002; Marino & Becker, 1977; Sobczak, Kula, & Danch, 2002; Zare, Hayatgeibi, Alivandi, & Ebadi, 2005). ELFs can affect the activity of brain neurons and thereby, interfere with brain waves as proved in extensive research on human and animal models. ELFs can reduce or increase the amplitude of different brain waves depending on their frequencies. Although, the magnitude and frequency of ELFs that decrease or increase the brain activity are not fully known, the organism’s response depends on duration of exposure to the field and effects of previous fields. Thus, the sensitivity of humans to magnetic fields differs by person and requires extensive research (Cook, Thomas, & Prato, 2002; Cvetkovic & Cosic, 2009; Cvetkovic, Fang, & Cosic, 2008).

On the other hand, different types of memories are part of the important functions of the nervous system. They are severely influenced by environmental factors and are able to change the mental state. Of these mental states, stress and anxiety can impair brain memory function (McEwen, Nasca, & Gray, 2015; Rostami et al., 2016). Psychologists and neurologists believe that hippocampus plays a key role in the formation of new memories of observed events (Richter-Levin & Akirav, 2000; Shahrivar, Moazedi, Rasekh, Almasi-Turk, & Roozbehi, 2014). Interestingly, ELFs can interact with the hippocampal neurons’ activity which may interfere with the ability of hippocampus for memory storage (Alkadhi, 2013; Rostami et al., 2016). This effect may be due to activation of stress system and possibly other hormones, which in fact shrinks the hippocampus size. In addition, increase in hippocampal glutamate system activity is shown to be involved in memory storage and therefore some investigators propose that stress hormones released during stressful events may induce glutamate system hyperactivity and reduce the cell size and connections within the hippocampus (Lucassen et al., 2014; McEwen et al., 2015; Tekieh et al., 2017).

The present research objective was to examine the effects of ELF with 1 Hz (as control group) and 12 Hz (as experimental group) frequencies with a magnitude of 0.7 μT on the cognitive, morphological, and hormonal changes in the rhesus macaque monkeys. For this purpose, Visual Working Memory (VWM), RMI images of the hippocampus, plasma level of melatonin and expression of glutamate N-Methyl-D-Aspartate (NMDA) receptor were evaluated in these animals. Melatonin (N-acetyl-5-methoxy tryptamine) is a hormone secreted from pineal gland and contributes to modulation of the sleep-wake cycle (the circadian rhythm). The short-term effects of melatonin variations may affect decision-making, memory, and learning in humans (Fukunaga, Horikawa, Shibata, Takeuchi, & Miyamoto, 2002; Ozdemir et al., 2005; Touitou & Selmaoui, 2012).

## 2. Methods

### 2.1. Animals

Four adult male rhesus macaques (Macaca mulatta), 4–5 years old with an average weight of 4 kg were used for this study. In this research, two monkeys were placed in the 12-Hz electromagnetic field (experimental group) and two others monkeys at 1-Hz electromagnetic field (control group). The animals received all needed vaccines (Hepatitis-B, HIV, and herpes). The monkeys were kept at the animal room laboratory of Neuroscience Research Center, Baqiyatallah University of Medical Sciences for 150 days for adaptation. The animals were kept in 12:12 hours dark/light cycle at room temperature (24°C±2°C) with adequate food and water (mal at 8, 12, and 16 O’clock and water was provided with scaled water nipples co ntainer in 1000 mL volume specifically designed for monkeys). All of the experiments were conducted according to the Baqyiatallah Medical University Medical Ethics Committee (No. 112-1394).

### 2.2. ELF exposure procedure

The ELF equipment can generate different frequencies from 1 to 300 Hz, made by Dr. Jafargholi laboratory, Amir Kabir University of Technology, Tehran, Iran. This generator can produce a magnetic field with the magnitude of 0.7 μT in 160-cm diameter circle field. The ELF exposure was conducted for each primate as follows: each primate was transferred to the shielded room in a 1×1×1 m Teflon cage. The cage was put 50 cm away from the ELF equipment and the wave generator became ON. The exposure was lasted 4 h/d for each animal.

### 2.3. Visual Working Memory Test

A VWM device (hidden behind a curtain) was designed for this test. The device included two opaque dishes (each dish with a window opening in one direction and rewards invisible to the primates). The two coated dishes were on a movable stand (Cook et al., 2002; Richter-Levin & Akirav, 2000). The animals were tested after 17 hours of fasting. The primates were transferred to the behavioral test room separately, and the test was carried out in two phases.

#### 2.3.1. Cognitive Behavior Test

The VWM device was placed in front of the cage and the primate’s favorite reward (peanut) was shown to it which was randomly placed in one of the dishes. The curtain was drawn so that the animal cannot watch the dishes for 30 seconds. After this time, the curtain was removed and the dishes was presented to the animal on a movable stand. Each animal was allowed to make only one attempt to pick the reward from one of the two dishes. In other words, the animal had to remember the dish with the reward, and if the animal opened the wrong container, it would be deprived of the reward. This test was carried out three times a day for 30 days.

#### 2.3.2. Second experiment phase

The VWM test was administered with a minor difference. The time of curtain covering was doubled (60 seconds instead of 30 seconds). Then, the dish was presented to the animal. This test was also repeated 3 times a day (Constantinidis & Procyk, 2004).

### 2.4. MRI imaging

Animals were fasting for 9 h and then anesthetized using ketamine hydrochloride (10 mg/kg). A 3T MRI device in the T1 and T2 phases with 3-mm sections of the axial, sagittal, and coronal regions (for better anatomic interpretation of the regions of concern). In the image interpretation phase, volumetric assessments of hippocampus were analyzed in Image J (Jaba, Shanthi, Singh, 2011).

### 2.5. Hormonal assays

The primates were put in the stabilized state of consciousness and 10 mL of blood was obtained from their femoral vain (popliteal artery) for determination of melatonin content. The blood samples were divided into two parts. The first part was centrifuged at 3000 rpm for 5 minutes at a temperature of 4°C and the serum content was isolated for melatonin measurement using melatonin ELISA kit (MyBioSource, USA).

### 2.6. Assays of NMDA receptors gene expression

The second part of the blood samples was used for cellular and molecular assays. After collecting the blood samples, its lymphocytes were isolated using the Ficoll solution in a centrifuge at 1500 rpm in for 5 minutes followed by another 15 minutes at 2500 rpm. The isolated lymphocytes were tested to determine expression of NMDA receptor genes using the PCR technique. To assess the impact of the ELF exposure on the expression of NAMD receptor gene, the semi-quantitative reverse transcriptase-polymerase chain reaction (semi-RT-PCR) was utilized. As described earlier, peripheral blood sample was collected from each animal in related time and the total mRNA was purified by the RNX-Plus kit (CinnaGen, Iran) in accordance with the manufacturer’s guideline. The quantity and quality of each isolated RNA was evaluated using the NanoDrop spectrophotometer (Thermos, USA) and agarose gel electrophoresis, respectively. After that, to synthesize cDNA from each sample, Bioneer kit (Takara, Japan) was applied. Briefly, 100 ng of each RNA sample was converted to cDNA by the Master Mix containing M-MLV Reverse Transcriptase, random hexamers, oligo (dT) and related buffer. Finally, the NAMD-2A gene expression was detected using PCR and related specific primer set. The mRNA expression of β-actin was assessed as an internal control. All PCR reactions were performed in a thermal cycler (Techne, UK) containing 1.5 μL cDNA, 0.2 mM of the deoxyribonucleoside tri-phosphates (dNTPs), 2.5 mM MgCl_2_, 10 pmol of each primers and 1.5 U of Taq DNA Polymerase (CinnaGen, Iran). PCR program consisted of 6 min initial denaturation at 94°C, 35 cycles of 45 s at 95°C, 45 s at 58°C for NMDA and β-actin and 56°C for NMDA 2A and 1 min at 72°C followed by 7 min final extension at 72°C. To measure the density of amplicons, each PCR product was run on 2% agarose gel electrophoresis, stained by ethidium bromide and visualized under UV gel document. Finally, the density of each product band was measured by Image J (Hillmann, Ramdas, Multanen, Norman, & Harmon, 2000; Mahmoodzadeh Hosseini, Soleimanirad, Mehdizadeh Aghdam, Amin, & Fooladi, 2015).

### 2.7. Analysis method

The study method is descriptive because the number of samples was low. Two types of comparisons were made in this study. First, the monkeys were compared with themselves (before and after radiation). That is, monkeys exposed to 12 Hz and monkeys exposed to 1 Hz ELF. Second, because monkeys exposed to 1 Hz did not show much changes they were introduced as the control group and compared with monkeys exposed to 12 Hz as the experimental group.

## 3. Results

**Figure 1:**
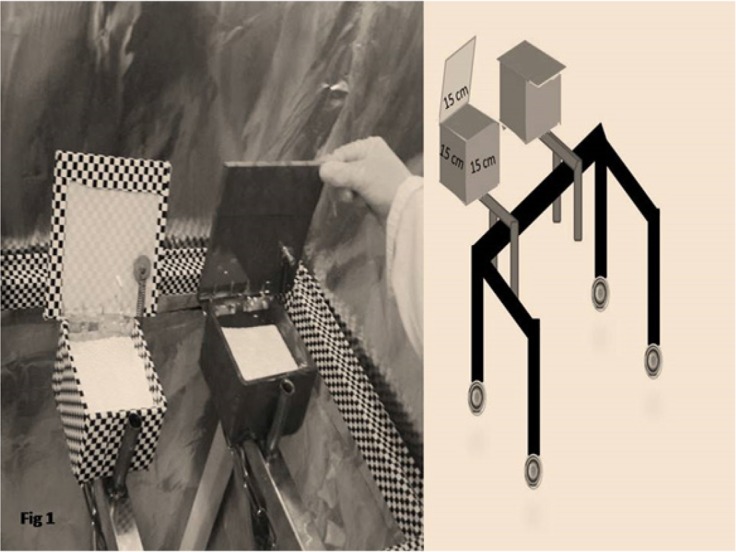
Plexiglass coated dishes (containing invisible reward)

Results show the percentage of correct answers of VWM test of the primates after radiation with ELF waves. It revealed that in the B–F (the experiment) monkeys (exposed to the 0.7 μT field at 12 Hz), VWM scores (with a hidden reward) changed considerably following the radiation. However, in the D–E monkeys, which was exposed to the 0.7 μT field at 1 Hz (control group), VWM did not change considerably (Figure 1) (Table 1).

**Table 1:** The percentages of correct answers of VWM before and after irradiation in the monkeys^*^

**Visual Working Memory**		

**Monkeys Code Wave Frequency**	**30 s Delay / Non Visible Correct Response**	**60 s Delay / Non Visible Correct Response**
B–F	Per 51 / Post 62	Per 36 / Post 45

12 HZ		
D–E	Per 30 / Post 31	Per 24/ Post 23

1 HZ		

* Visual working memory decreased significantly in the 30- and 60-second periods as compared to the pre-radiation state.

**Figure 2:**
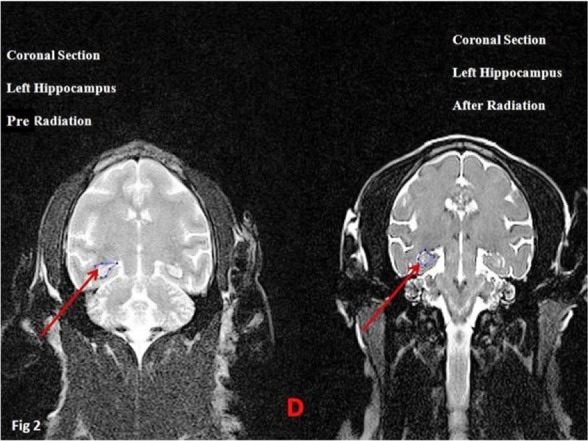
Volumetric analysis of the left hippocampus of primates (D-B) Arrows show the hippocampus.

**Figure 3:**
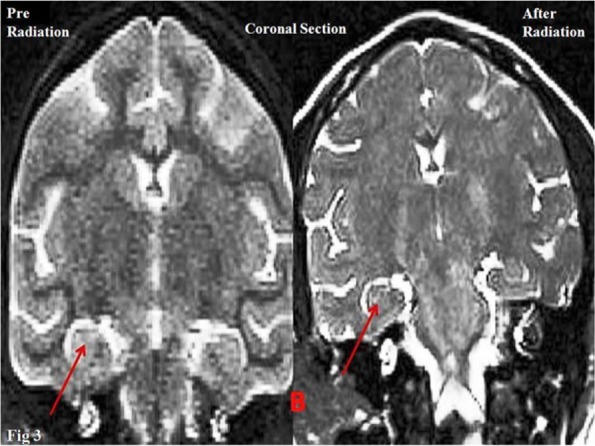
Volumetric analysis of the left hippocampus of primates (D-B) Arrows show the hippocampus.

Results of anatomical assays (MRI) at 12 and 1 Hz (The wavelength of 1 Hz was used as control) frequencies using the 0.7 μT field did not reveal significant changes in volumetric properties of the left hippocampus in the coronal or axial section (Figures 2, 3) (Table 2).

**Table 2:** Changes in the volume of the left hippocampus after radiation with 1 Hz (D–E) and 12 Hz (B–F)

**Monkey**	**Coronal Section Left Hippocampus Volume (mm)^3^ Pre-radiation**	**Coronal Section Left Hippocampus Volume (mm)^3^ Post-radiation**

**Codes**		
B–F	293.9	316.2

12 HZ		
D–E	345.225	353.345

1 HZ		

No significant difference was observed.

**Figure 4:**
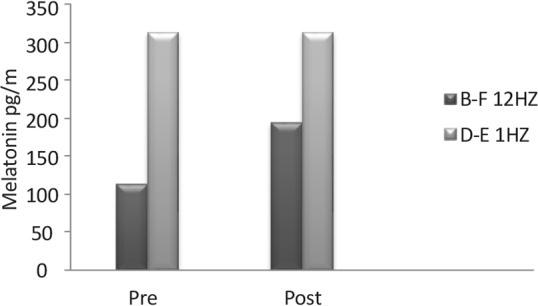
N-methyl-D-aspartate receptor gene expression in the 1 Hz (D–E) and 12 Hz (B–F) wavelengths exposed animals The expression of these receptors’ gene was higher in the 12 Hz exposed animals than the control animals.

Plasma melatonin increased following the radiation of the 12 Hz wave with 0.7 μT field magnitude in the experimental primates and remained unchanged under the impact of the 1 Hz radiation in the control group. The recovery of both primates involved restoration of their states to the pre-radiation phase (Figure 4).

**Figure 5:**
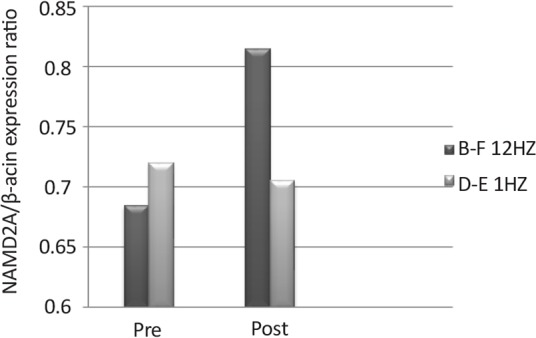
N-Methyl-D-Aspartate receptor gene expression in the 1 HZ(D–E) and 12HZ(B–F) wavelengths exposed animals The expression of these receptors’ gene was higher in the 12 HZ animals than the control animals.

Effects of radiation with the 12 Hz and 1 Hz frequencies (1 Hz frequency was used as control) with 0.7 μT field on expression of the NMDA receptor in the control and experiment primates showed that changes of expression of the NMDA gene under the impact of the 12 Hz wave and the 0.7 μT field increased significantly in the experimental primates following radiation. However, expression of NMDA gene under the impact of the 1 Hz wave and 0.7 μT fields changes slightly in the control group following radiation. The recovery of both primates involved restoration of their states to the pre-radiation phase (Figure 5).

## 4. Discussion

Our experiments indicate that exposure to the low-frequency (12 Hz) electromagnetic wave with 0.7 μT magnetic field magnitude may have a significant effect on cognitive functions changes, as shown in VWM decline in primates. Besides this memory decline, results showed a decrease in plasma melatonin concentration and an increase in NMDA receptor gene expression. These results may reveal hazardous features of ELF on living organisms, especially the primates.

Previous studies indicate that the ELF effect can be various with respect to the wave frequencies and the radiation period as well as biological and physiological properties of humans and animals (Ross et al., 2015; Salford & Nittby, 2012). In this regard, it is shown that magnetic field with a frequency of 50 Hz impair memory and learning in human (Sakhnini et al., 2014; Salford & Nittby, 2012). Results of the other researchers have also revealed that ELF waves with 10 and 30 Hz frequencies and field magnitude of 2 μT improves memory and learning in rats (An et al., 2015; Casile, 2013; D’Angelo, Costantini, Kamal, & Reale, 2015; Rimbach et al., 2014; Sakhnini et al., 2014).

Working memory is an important and fundamental cognitive process that forms the basis of thinking and learning also it is associated with storage and processing of information in the mind. Results of cognitive tests show the percentage of correct answers significantly increased after irradiation with 12 Hz frequency both at 30 s and 60 s schedules.

In addition, as shown in Table 1, the VWM test scores were particularly enhanced in the animals exposed to the 12-Hz ELF, therefore the exposure may be strong enough to interact with the areas of the animals’ brains that increase their memory capacity. We hold that this enhancement may occur in the hippocampus as it is shown in several animal and human studies (Keller & Roberts, 2009; Jaba, Shanthi, & Singh, 2011; Tae et al., 2008; Tekieh et al., 2017). However, it is interesting that our results in MRI studies did not support our hypothesis. To date, there is no discussion about this phenomenon but one may conclude that the effects of the 12-Hz ELF exposure may affect other brain areas. This idea must be studied in the future research.

In another part of the present study, the effect of 12-Hz ELF exposure was examined on the melatonin hormone. Melatonin is considered as the sleep and wake hormone released from pineal gland during dark period (Baydas et al., 2005; D’Angelo et al., 2015; Pandiperumal et al., 2008). Our data indicate that melatonin plasma concentration is elevated after 12-Hz ELF exposure. The increased plasma melatonin level may be responsible for the increased animals’ VWM scores. In this regard, several studies support that melatonin can increase brain cognitive function via increase in the beta wave in EEG (Baharara & Zahedifar, 2012; Baydas et al., 2005; Hampton et al., 2005; Lingnau, Gesierich, & Caramazza, 2009). However, it is too soon to draw conclusion about the hormonal effect on VWM.

The hippocampus-subiculum path is important in learning, memory process; also Long-Term Potentiation (LTP) mechanism is important for the formation of some forms of memory and learning. In the visual cortex, the Long-Term Depression (LTD) and LTP points display many mechanisms with synaptic plasticity that associate with the sense of sight. LTP is related with the visual cortex that is dependent on the NMDA receptor channels with their significant role in learning and memory (Lynch, 2004; Morris, 2013; Nakazawa, McHugh, Wilson, & Tonegawa, 2004; Newcomer, Farber, & Olney, 2000).

Finally, our result indicate that N-Methyl-D-Aspartate glutamate receptors (NMDA) gene expression was elevated after 12-Hz ELF exposure. This finding may indicate that by an unknown mechanism(s), ELF can increase the gene expression as indicated by the increment in the gene expression in our study. This part of the study may also support the VWM high scores in these animals as well.

In conclusion, 12-Hz ELF exposure enhances the brain cognitive function in the rhesus monkeys as indicated by the increase in VWM test scores. This improvement may be due to increase in plasma melatonin hormone and or increase in NMDA glutamate receptor gene expression. However, the potential clinical use of these findings would be a suitable topic for the future experiments.

## Ethical Considerations

### Compliance with ethical guideline

All of the experiments were conducted according to the Baqyiatallah Medical University Medical Ethics Committee (No. 112-1394).
